# “Neighbohod Vulnerability and Disability in First Episode of Psychosis”.

**DOI:** 10.1192/j.eurpsy.2024.607

**Published:** 2024-08-27

**Authors:** I. Leal, A. Izquierdo, M. Cabello

**Affiliations:** ^1^psiquiatria, HOSPITAL DE LA PRINCESA, MADRID; ^2^psiquiatria, Universidad autonoma de madrid, spain, Spain

## Abstract

**Introduction:**

Neighborhood socioeconomic status seems to be related to functioning in patients with first episode of psychosis

(FEP).

**Objectives:**

The present study aimed to assess if neighborhood vulnerability and risk of social exclusion could predict functional outcomes in people with FEP after controlling for other key variables identified in previous literature.

**Methods:**

A total of 137 patients with FEP (DSM-IV-TR criteria) and 90 controls comprised the study sample from February 2013 to May 2019. Functioning was assessed with the WHO Disability Assessment Schedule. Neighborhood vulnerability was measured using a multidimensional socioeconomic deprivation index; data for the index were collected by the Madrid City Council and based on the participant’s home address. Multilevel mixed-effects regression analyses were conducted to estimate the effects of neighborhood vulnerability on functioning.

**Results:**

Our results show that FEP patients could be more vulnerable to the effects of neighborhood-level characteristics than healthy controls (B = 1,570.173; z = 3.91; Pc .001). In addition, our findings suggest that higher neighborhood vulnerability is related to greater functional disability in people with FEP, after controlling for other relevant confounders (B = 1,230.332; 2=2.59; P=.010). based on the participant’s home address. Multilevel mixed-effects regression analyses were conducted to estimate the effects of neighborhood vulnerability on functioning.

**Results:**

Our results show that FEP patients could be more vulnerable to the effects of neighborhood-level characteristics than healthy controls (B = 1,570.173; z = 3.91; Pc 001). In addition, our findings suggest that higher neighborhood vulnerability is related to greater functional disability in people with FEP, after controlling for other relevant confounders (B = 1,280.332; z=2.59; P= 010).

**Conclusions:**

These results highlight the importance of incorporating contextual factors into assessment of patients with FEP, since psychosocial difficulties observed In these patients could be partially related to the quality of neighborhood social-related resources.

**Disclosure of Interest:**

None Declared

